# Unwinding new therapeutic opportunities in rhabdomyosarcoma: the role of RNA helicase DDX5

**DOI:** 10.3389/fcell.2025.1662619

**Published:** 2025-08-29

**Authors:** Valeria Bianconi, Chiara Mozzetta

**Affiliations:** Department of Biology and Biotechnologies “Charles Darwin”, Sapienza University of Rome, Rome, Italy

**Keywords:** RNA helicase, DEAD-box, rhabdomyosarcoma, cancer therapy, tumor proliferation

## Abstract

Rhabdomyosarcoma (RMS) is one of the most common soft tissue tumors in children and is primarily classified into two subtypes: alveolar (ARMS) and embryonal (ERMS). Among these, ARMS is the more aggressive form, often driven by chromosomal translocations that give rise to PAX3/7-FOXO1 fusion proteins, which act as oncogenic transcription factors. Despite advancements in treatment and improved survival rates over recent years, effective and targeted therapies for RMS remain a significant clinical challenge. A family of proteins known as the DEAD-box RNA helicases plays a critical role in RNA metabolism as well as in a variety of cellular processes beyond RNA regulation. Among them, DDX5 has emerged as a protein of particular interest. Aberrant expression and functional alterations of DDX5 have been reported in multiple cancers, including RMS, where its overexpression is associated with enhanced tumor growth and cancer cell proliferation. In this review, we highlight recent discoveries that position DDX5 as a promising therapeutic target in RMS, focusing on its oncogenic functions and its contribution to tumorigenesis and cancer progression.

## Introduction

Rhabdomyosarcoma (RMS) is the most frequently diagnosed soft tissue sarcoma in children and adolescents, accounting for approximately 40% of pediatric soft tissue sarcomas, with an incidence of 4.5 cases per million per year (Ognjanovic et al., 2009). RMS is believed to arise from mesenchymal progenitor cells committed to the skeletal muscle lineage (Skapek et al., 2019), and it is broadly classified into two major subtypes—alveolar (ARMS) and embryonal (ERMS)—which differ significantly in their histological, molecular, and clinical characteristics (Wachtel and Schäfer, 2018).

The alveolar subtype (ARMS), more common in older children and adolescents, is predominantly characterized by chromosomal translocations that generate PAX3/7-FOXO1 fusion oncoproteins. These fusions are major oncogenic drivers but are notoriously difficult to target pharmacologically (Wachtel and Schäfer, 2018). Approximately 20% of ARMS cases, however, lack these fusions (Hettmer et al., 2014; Barr et al., 2002) and are nearly indistinguishable from ERMS at the molecular level (Williamson et al., 2010). Fusion-positive ARMS tumors are associated with higher metastatic potential, resistance to therapy, and poorer clinical outcomes compared to their fusion-negative counterparts, and are thus considered the more aggressive form of RMS (Hettmer et al., 2014; Williamson et al., 2010).

In contrast, ERMS typically presents in younger children and shows morphological features resembling developing skeletal muscle. ERMS lacks specific chromosomal translocations but displays widespread genomic instability (Sun et al., 2015) and is generally associated with a more favorable prognosis.

Current RMS treatment follows a multimodal strategy combining surgery, chemotherapy, and radiotherapy, tailored according to histological subtype and disease stage (Łomiak et al., 2023). Recent advances include the use of proton beam therapy (Dong et al., 2023), immune checkpoint inhibitors (Davis et al., 2020), and CAR-T cell-based therapies (Tian et al., 2023). Despite these improvements, treatment resistance and disease recurrence remain major clinical challenges, underscoring the urgent need for novel therapeutic targets and robust diagnostic biomarkers.

Among the emerging molecular players in cancer biology are members of the DEAD-box RNA helicase family, highly conserved enzymes involved in virtually all aspects of RNA metabolism. Beyond their canonical roles, several DEAD-box helicases have been implicated in cancer progression, where their overexpression is associated with increased tumor proliferation and survival.

Recent studies have begun to elucidate the role of DEAD-box helicase 5 (DDX5) in RMS. DDX5 is significantly upregulated in ARMS and supports tumor cell proliferation and survival both *in vitro* and *in vivo*, partly through its interaction with the histone methyltransferase EHMT2 (Gualtieri et al., 2022). Moreover, co-depletion of DDX5 and the m6A reader protein YTHDC1 leads to impaired proliferation in RMS cell models, suggesting an oncogenic role for DDX5 in this context (Dattilo et al., 2023).

In this review, we summarize and critically evaluate current knowledge regarding DDX5 function in RMS. We highlight the molecular mechanisms by which DDX5 contributes to RMS pathogenesis and discuss its potential as a novel therapeutic target.

## Physiological and oncogenic functions of DDX5

DDX5, also known as p68, is a highly conserved member of the DEAD-box RNA helicase family, defined by the hallmark Asp-Glu-Ala-Asp (DEAD) motif. It plays multifaceted roles in both physiological processes and cancer pathogenesis. In normal cellular physiology, DDX5 contributes significantly to RNA metabolism, including pre-mRNA splicing, microRNA (miRNA) processing, RNA export, and mRNA decay (Linder and Jankowsky, 2011). Beyond its RNA-related functions, DDX5 also acts as a transcriptional co-activator for key regulators such as p53 (Bates et al., 2005) and β-catenin (Yang et al., 2006; Wang et al., 2015).

**FIGURE 1:**
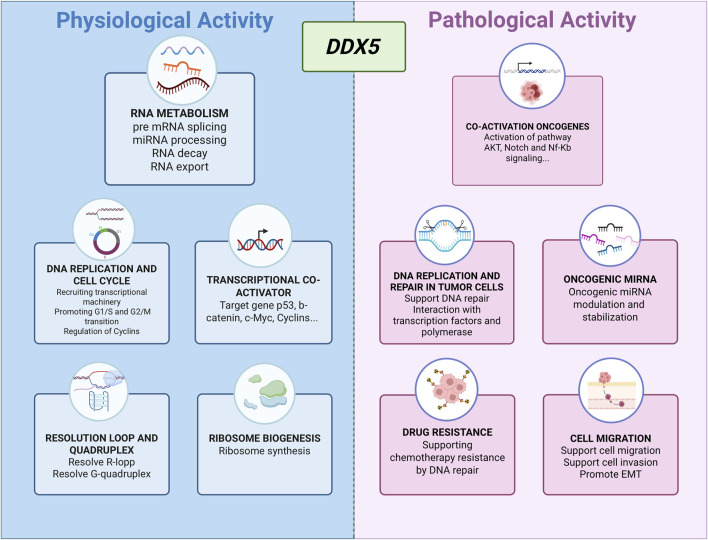
Overview of the diverse functions of DDX5 in physiological and pathological contexts.

DDX5 participates in DNA replication and cell cycle progression by facilitating the expression of replication machinery components, recruiting transcriptional complexes, and regulating cyclin levels to support G1/S and G2/M transitions (Mazurek et al., 2012; Nicol et al., 2013; LeGrand et al., 2019; Sergeeva and Zatsepin, 2021; Zhang et al., 2021). It also contributes to ribosome biogenesis (Saporita et al., 2011), the resolution of RNA-DNA hybrids (Mersaoui et al., 2019; Yu et al., 2020), and the unwinding of G-quadruplex DNA structures (Wu et al., 2019) (Figure 1).

Consistent with its role as a versatile RNA helicase, DDX5 has emerged as a multifunctional oncogenic driver in both solid and hematologic malignancies, integrating upstream signaling inputs with downstream transcriptional and post-transcriptional programs that sustain tumor growth, survival, and therapy resistance. In colorectal cancer (CRC), for example, DDX5 directly interacts with β-catenin to act as a transcriptional co-activator, upregulating FOXM1 and promoting tumor progression (Tabassum et al., 2023). PDGF stimulation in CRC cells further exemplifies its responsiveness to extracellular cues: phosphorylation of DDX5 enables binding to β-catenin and displacement of Axin, driving β-catenin nuclear translocation through a Wnt-independent route and activating EMT-related gene expression (Yang et al., 2006). In breast cancer, DDX5 facilitates the G1–S phase transition by recruiting RNA polymerase II to E2F-regulated promoters, thereby increasing the expression of DNA replication factors (Mazurek et al., 2012). In non-small cell lung cancer (NSCLC), DDX5 overexpression sustains Wnt/β-catenin signaling, promotes nuclear accumulation of β-catenin, and co-activates cyclin D1 and c-Myc expression (Li et al., 2021; Wang et al., 2015), while in thyroid cancer it enhances metastatic potential through interaction with E2F1 (Yuan et al., 2020).

In prostate cancer, DDX5 links oncogenic transcription to genome stability maintenance. It contributes to tumor progression by stabilizing oncogenic mRNAs and by participating in nucleotide excision repair through interactions with Ku70/Ku80, NF45/NF90, TFIIH, and RFC complexes (Le et al., 2023), with its stability in castration-resistant disease further maintained by heat shock protein 27. In hematologic malignancies, the oncogenic output of DDX5 appears context-dependent: in T-cell acute lymphoblastic leukemia (T-ALL) it associates with the MAML1 coactivator of Notch signaling but is dispensable for proliferation, whereas in acute promyelocytic leukemia (APL) and acute myeloid leukemia (AML), where expression levels are higher, DDX5 activity is essential for cell survival.

Together, these findings underscore how DDX5 functions as a signaling-responsive regulator whose impact depends on tumor context, upstream modifiers, and expression level—features that may critically influence responsiveness to targeted inhibition (Wu et al., 2020; Xu et al., 2022) (Figure 1). Different small-molecule inhibitors have been developed to inhibit DDX5 function via distinct mechanisms, including helicase activity inhibition, disruption of protein–protein interactions, and protein destabilization. Among them, FL118 has shown potent antitumor effects by reducing DDX5 expression in models of pancreatic and colorectal cancer, as well as chronic myeloid leukemia (Ling et al., 2022; Ling et al., 2024; Takeda et al., 2024). Although FL118 has been proposed to interfere with the RNA helicase activity of DDX5 (Takeda et al., 2024), its predominant mechanism of action appears to involve protein destabilization (Ling et al., 2022). Indeed, FL118 binds with DDX5, thereby suppressing its phosphorylation and inducing its degradation by the ubiquitin–proteasome system (Ling et al., 2022). Another promising compound, RX5902 (Supinoxin), binds phosphorylated DDX5 (Tyr593), blocking its ATPase activity and interaction with βcatenin. RX5902 has demonstrated the ability to inhibit proliferation and EMT *in vitro*, and reduce tumor growth in xenograft models of breast cancer (Kost et al., 2015; Capasso et al., 2019; Tentler et al., 2020). It is currently undergoing Phase I–II clinical trials for solid tumors, including triple-negative breast cancer.

Despite these promising developments, no DDX5-targeted strategy has yet been applied to Rhabdomyosarcoma. Given its pleiotropic roles in promoting tumor growth and resistance, further exploration of DDX5 in RMS is warranted, both to better understand its biological relevance and to evaluate its potential as a therapeutic target.

## DDX5 as a key regulator in fusion-positive rhabdomyosarcoma

**FIGURE 2:**
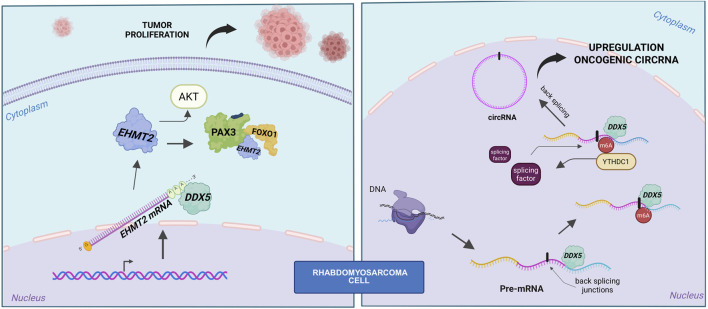
Role of DDX5 in fusion-positive RMS. Left panel: schematic representation of the DDX5–PAX3-FOXO1–EHMT2 axis, showing that DDX5 stabilizes *EHMT2* mRNA. *EHMT2* in turn sustains both AKT activity and PAX3-FOXO1 stability. Right panel: DDX5 cooperates with YTHDC1 to promote back-splicing and circRNA biogenesis.

In alveolar rhabdomyosarcoma (ARMS), particularly in fusion-positive cases (FP-RMS), DDX5 plays a pivotal role in sustaining tumor growth and survival. Its activity in this context appears to be mediated through two complementary mechanisms: regulation of post-transcriptional gene expression—most notably by stabilizing the histone methyltransferase EHMT2—and modulation of the fusion oncoprotein PAX3-FOXO1 (Gualtieri et al., 2022). In parallel, DDX5 also contributes to shaping the non-coding RNA landscape, including circular RNA (circRNA) production (Dattilo et al., 2023) (Figure 2).

### The DDX5–EHMT2–PAX3-FOXO1 axis: a tumor-specific circuit driving fusion-positive RMS

DDX5 expression has been found significantly elevated in FP-RMS tumors compared to both normal skeletal muscle and fusion-negative RMS (Gualtieri et al., 2022), suggesting a pathogenic role. In support of this, functional studies have shown that silencing of DDX5 leads to a marked reduction in FP-RMS cell proliferation and consequent induction of apoptosis. Importantly, this effect appears to be tumor-specific, as DDX5 depletion in human skeletal muscle myoblasts (HSMMs) does not impact their viability or proliferation (Gualtieri et al., 2022).

Further evidence for DDX5 as a potential therapeutic target comes from its phosphorylation status in RMS. Hyperphosphorylated DDX5 (p-DDX5) is enriched in FP-RMS, and pharmacological targeting of this form with RX-5902—a small molecule that blocks DDX5–β-catenin interaction—has shown promising anti-tumor activity. In FP-RMS cells, RX-5902 treatment results in decreased nuclear β-catenin accumulation, dose-dependent growth arrest, and induction of apoptosis, while sparing non-transformed muscle cells (Gualtieri et al., 2022).

Transcriptomic analysis of DDX5-depleted FP-RMS cells revealed significant downregulation of genes involved in RAS signaling, particularly the PI3K-AKT pathway, suggesting that DDX5 supports oncogenic signaling cascades. Mechanistically, DDX5 was shown to bind preferentially to the 3′untranslated regions (UTRs) of mRNAs encoding chromatin and transcriptional regulators, including *EHMT2*. Knockdown of DDX5 reduced the spliced, mature *EHMT2* transcript without altering levels of the unspliced form, indicating a role in mRNA stabilization rather than transcriptional initiation or splicing regulation (Gualtieri et al., 2022).

A functional axis involving DDX5, EHMT2, and PAX3-FOXO1 has been proposed, where DDX5 not only stabilizes EHMT2 mRNA but also forms an RNA-dependent complex with both EHMT2 and PAX3-FOXO1. Depletion of either DDX5 or EHMT2 leads to a reduction in PAX3-FOXO1 protein levels without affecting its mRNA expression, supporting a post-transcriptional regulatory mechanism. Although no direct methylation of PAX3-FOXO1 by EHMT2 was detected, its enzymatic activity appears essential for maintaining PAX3-FOXO1 protein stability (Gualtieri et al., 2022).

The relevance of this axis is further supported by the observation that EHMT2 knockdown downregulates canonical PAX3-FOXO1 target genes, including MYOD1 and MYOGENIN. Disruption of DDX5 or EHMT2 function also suppresses PI3K-AKT signaling, reduces proliferation, and induces apoptosis in FP-RMS cells. Restoration of EHMT2 in DDX5-silenced cells partially rescues cell growth, positioning DDX5 functionally upstream in this regulatory network (Gualtieri et al., 2022).

Finally, *in vivo* experiments using xenograft models demonstrated that DDX5 depletion results in significantly impaired tumor growth, accompanied by reduced expression of both EHMT2 and PAX3-FOXO1. Together, these findings define a tumor-specific DDX5–EHMT2–PAX3-FOXO1 axis critical for FP-RMS maintenance (Gualtieri et al., 2022). Given that RX-5902 is already under clinical investigation in other solid tumors, targeting DDX5 may offer a viable and selective therapeutic strategy for fusion-positive rhabdomyosarcoma.

### DDX5 fuels circRNA biogenesis in RMS

DDX5 has recently been identified as a central player in a novel oncogenic RNA regulatory pathway in RMS, where it cooperates with the m^6^A reader YTHDC1 to promote the expression of a specific subset of circular RNAs (circRNAs) (Dattilo et al., 2023). Among RNA modifications, m^6^A is one of the most extensively characterized, with roles extending well beyond circRNA biogenesis. For instance, studies on circ-ZNF609 have demonstrated that m^6^A marks, deposited by methyltransferases such as METTL3 and recognized by YTHDC1, can influence whether an RNA transcript is processed into a circular or linear form, thereby modulating both its biogenesis and translational potential (Di et al., 2020). Moreover, m^6^A modifications have been shown to trigger the translation of circRNA-derived proteins under environmental stress conditions, underscoring a functional role for these modified circRNAs beyond RNA stability (Yang et al., 2017). Supporting this, the m^6^A-modified circMYO1C, derived from the *MYO1C* gene, has recently been identified as upregulated in pancreatic ductal adenocarcinoma (PDAC), where it appears to contribute to oncogenesis via an m^6^A-dependent mechanism (Guan et al., 2023).

CircRNAs themselves have been implicated in oncogenic processes in RMS (Rossi et al., 2021), and the study by Dattilo and colleagues provides important mechanistic insights by demonstrating that YTHDC1 and DDX5 act cooperatively to drive the biogenesis of tumor-promoting circRNAs in this context (Dattilo et al., 2023). Intriguingly, RMS cell lines exhibit a global increase in circRNA abundance relative to normal myoblasts, a phenomenon not accompanied by corresponding changes in linear RNA levels, suggesting a tumor-specific shift favoring circular isoform production. This observation prompted investigation into the m^6^A machinery, revealing upregulation of key components such as METTL3, METTL14, and YTHDC1 in RMS cells (Dattilo et al., 2023). Among these factors, YTHDC1 emerges as a pivotal regulator of circRNA biogenesis: its depletion in RMS cells leads to a significant drop in circRNA levels without affecting overall transcription, highlighting its role in facilitating back-splicing (Dattilo et al., 2023). DDX5, previously reported to interact with METTL3 (Zhao et al., 2018), was identified as a key co-regulator in this process. This connection aligns with emerging evidence linking DDX5 to circRNAs function (He et al., 2024) and biogenesis in other cancers, such as gastric cancer, where DDX5-driven circRNAs promote tumor growth and invasion (Wang et al., 2023).

Mechanistically, DDX5 interacts with YTHDC1 independently of RNA, and knockdown of either factor alone—or in combination—markedly reduces circRNA abundance and causes G2/M cell cycle arrest (Dattilo et al., 2023). Notably, classical DDX5 targets, including *MYC* and *CCND1*, remain unaffected by DDX5 depletion, indicating a distinct, circRNA-specific role. Most circRNAs downregulated upon DDX5 knockdown overlap with those regulated by YTHDC1, supporting a shared regulatory circuit that operates independently of transcriptional output, RNA export, or stability (Dattilo et al., 2023). DDX5 appears to bind structured, GC-rich regions near back-splicing junctions in precursor transcripts (Dattilo et al., 2023), where its helicase activity likely remodels RNA structure to expose sites for m^6^A modification. While METTL3 depletion—but not DDX5 loss—reduces m^6^A signals, YTHDC1 may subsequently recruit splicing factors to these modified regions, jointly enhancing circRNA production.

In summary, this body of work reveals a previously unrecognized RNA regulatory mechanism in RMS whereby m^6^A modification and the coordinated activity of YTHDC1 and DDX5 promote the biogenesis of oncogenic circRNAs. The dependency of RMS cell proliferation on these factors highlights their potential as therapeutic targets, with implications that may extend to other malignancies harboring similar regulatory pathways.

## Concluding remarks

The multifaceted oncogenic functions of the DEAD-box RNA helicase DDX5 across various malignancies have brought it into focus as a promising therapeutic target. In the context of rhabdomyosarcoma, and particularly in the fusion-positive (FP-RMS) subtype, DDX5 emerges as a central regulator of tumorigenesis, with implications for both disease progression and treatment. In this review, we highlighted key evidence positioning DDX5 at the crossroads of RMS cell survival and proliferation, operating through at least two distinct and complementary mechanisms. On one hand, DDX5 engages in a pro-survival regulatory axis with the histone methyltransferase EHMT2 and the fusion oncoprotein PAX3-FOXO1, stabilizing oncogenic transcripts and reinforcing signaling pathways such as PI3K-AKT. On the other, DDX5 cooperates with the m^6^A reader YTHDC1 to drive the biogenesis of a tumor-specific subset of circular RNAs (circRNAs), which further contribute to RMS pathogenesis.

These dual roles—spanning transcriptional co-activation, post-transcriptional regulation, and RNA remodeling—highlight DDX5 as a multifunctional hub in FP-RMS biology. Importantly, its tumor-specific phosphorylation status has enabled the development of targeted pharmacological inhibitors, such as RX-5902, which has already demonstrated preclinical efficacy and is undergoing evaluation in early-phase clinical trials for other cancers. This existing therapeutic groundwork offers a significant advantage for the repurposing and clinical translation of DDX5-targeting strategies in RMS.

In summary, the body of evidence reviewed here underscores DDX5 as a promising and selective therapeutic target in RMS, with particular relevance to the alveolar subtype. Moving forward, bridging the remaining gaps in our understanding of its tumor-driving mechanisms and refining inhibitors for maximal efficacy and safety will be pivotal. With its tumor-restricted activity and far-reaching regulatory roles, DDX5 inhibition offers the prospect of reshaping therapeutic paradigms—not only in pediatric RMS, but across a spectrum of DDX5-dependent malignancies—paving the way for more precise, mechanism-based cancer therapies.

## References

[B1] Barr F. G. Qualman S. J. Macris M. H. Melnyk N. Lawlor E. R. Strzelecki D. M. (2002). Genetic heterogeneity in the alveolar rhabdomyosarcoma subset without typical gene fusions. Cancer Res. 62 (16), 4704–4710. 12183429

[B2] Bates G. J. Nicol S. M. Wilson B. J. Jacobs A. M. Bourdon J. C. Wardrop J. (2005). The DEAD box protein p68: a novel transcriptional coactivator of the p53 tumour suppressor. EMBO J. 24 (3), 543–553. 10.1038/sj.emboj.7600550 15660129 PMC548656

[B3] Capasso A. Bagby S. M. Dailey K. L. Currimjee N. Yacob B. W. Ionkina A. (2019). First-in-Class Phosphorylated-p68 inhibitor RX-5902 inhibits β-Catenin signaling and demonstrates antitumor activity in triple-negative breast cancer. Mol. Cancer Ther. 18 (11), 1916–1925. 10.1158/1535-7163.MCT-18-1334 31488700 PMC6825586

[B6] Dattilo D. Di Timoteo G. Setti A. Giuliani A. Peruzzi G. Beltran Nebot M. (2023). The m6A reader YTHDC1 and the RNA helicase DDX5 control the production of rhabdomyosarcoma-enriched circRNAs. Nat. Commun. 14 (1), 1898–15. 10.1038/s41467-023-37578-7 37019933 PMC10076346

[B7] Davis K. L. Fox E. Merchant M. S. Reid J. M. Kudgus R. A. Children’s Oncology Group ADVL1412 Investigators (2020). Nivolumab in children and young adults with relapsed or refractory solid tumours or lymphoma (ADVL1412): a multicentre, open-label, single-arm, phase 1-2 trial. Lancet Oncol. 21 (4), 541–550. 10.1016/S1470-2045(20)30023-1 32192573 PMC7255545

[B8] Di T. G. Dattilo D. Centrón-Broco A. Colantoni A. Guarnacci M. Rossi F. (2020). Modulation of circRNA metabolism by m6A modification. Cell Rep. 31 (6), 107641. 10.1016/j.celrep.2020.107641 32402287

[B9] Dong M. Wu J. Wu R. Wang D. Liu R. Luo H. (2023). Efficacy and safety of proton beam therapy for rhabdomyosarcoma: a systematic review and meta-analysis. Radiat. Oncol. 18 (1), 31. 10.1186/s13014-023-02223-6 36805784 PMC9942395

[B10] Gualtieri A. Bianconi V. Renzini A. Pieroni L. Licursi V. Mozzetta C. (2022). The RNA helicase DDX5 cooperates with EHMT2 to sustain alveolar rhabdomyosarcoma growth. Cell Rep. 40 (9), 111267. 10.1016/j.celrep.2022.111267 36044855

[B11] Guan H. Tian K. Luo W. Li M. (2023). m6A-modified circRNA MYO1C participates in the tumor immune surveillance of pancreatic ductal adenocarcinoma through m6A/PD-L1 manner. Cell Death Dis. 14, 120. 10.1038/s41419-023-05570-0 36781839 PMC9925427

[B12] He S. Bai J. Zhang L. Yuan H. Ma C. Wang X. (2024). Superenhancer-driven circRNA Myst4 involves in pulmonary artery smooth muscle cell ferroptosis in pulmonary hypertension. iScience 27 (10), 110900. 10.1016/j.isci.2024.110900 39351203 PMC11440257

[B13] Hettmer S. Li Z. Billin A. N. Barr F. G. Cornelison D. D. Ehrlich A. R. (2014). Rhabdomyosarcoma: current challenges and their implications for developing therapies. Cold Spring Harb. Perspect. Med. 4 (11), a025650. 10.1101/cshperspect.a025650 25368019 PMC4208704

[B14] Kost G. C. Yang M. Y. Li L. Zhang Y. Liu C. Y. Kim D. J. (2015). A novel anti-cancer agent, 1-(3,5-Dimethoxyphenyl)-4-[(6-Fluoro-2-Methoxyquinoxalin-3-yl)Aminocarbonyl] piperazine (RX-5902), interferes with β-Catenin function through Y593 Phospho-p68 RNA helicase. J. Cell Biochem. 116 (8), 1595–1601. 10.1002/jcb.25113 25649741

[B15] Le T. K. Cherif C. Omabe K. Paris C. Lannes F. Audebert S. (2023). DDX5 mRNA-targeting antisense oligonucleotide as a new promising therapeutic in combating castration-resistant prostate cancer. Mol. Ther. 31 (2), 471–486. 10.1016/j.ymthe.2022.08.005 35965411 PMC9931527

[B16] Legrand J. M. D. Chan A. L. La H. M. Rossello F. J. Anko M. L. Fuller-Pace F. V. (2019). DDX5 plays essential transcriptional and post-transcriptional roles in the maintenance and function of spermatogonia. Nat. Commun. 10 (1), 2278. 10.1038/s41467-019-09972-7 31123254 PMC6533336

[B17] Li F. Fountzilas C. Puzanov I. Attwood K. M. Morrison C. Ling X. (2021). Multiple functions of the DEAD-Box RNA helicase, DDX5 (p68), make DDX5 a superior oncogenic biomarker and target for targeted cancer therapy. Am. J. Cancer Res. 11 (10), 5190–5213. 34765320 PMC8569338

[B19] Linder P. Jankowsky E. (2011). From unwinding to clamping - the DEAD box RNA helicase family. Nat. Rev. Mol. Cell Biol. 12 (8), 505–516. 10.1038/nrm3154 21779027

[B20] Ling X. Wu W. Aljahdali I. A. M. Liao J. Santha S. Fountzilas C. (2022). FL118, acting as a 'molecular glue degrader', binds to dephosphorylates and degrades the oncoprotein DDX5 (p68) to control c-Myc, survivin and mutant kras against colorectal and pancreatic cancer with high efficacy. Clin. Transl. Med. 12 (5), e881. 10.1002/ctm2.881 35604033 PMC9126027

[B21] Ling X. Wu W. Yan L. Curtin L. Farrauto M. M. Sexton S. (2024). Clinically and orally compatible formulation-manufactured DDX5 (p68)-targeting molecular glue FL118 products exhibit low toxicity but high efficacy against human cancer. J. Pharm. Anal. 14 (11), 101001. 10.1016/j.jpha.2024.101001 39759975 PMC11696646

[B22] Łomiak M. Świtaj T. Spałek M. Radzikowska J. Chojnacka M. Falkowski S. (2023). Diagnosis and treatment of rhabdomyosarcomas. Oncol. Clin. Pract. 19 (4), 250–279. 10.5603/OCP.2022.0034

[B24] Mazurek A. Luo W. Krasnitz A. Hicks J. Powers R. S. Stillman B. (2012). DDX5 regulates DNA replication and is required for cell proliferation in a subset of breast cancer cells. Cancer Discov. 2 (9), 812–825. 10.1158/2159-8290.CD-12-0116 22750847 PMC3440546

[B25] Mersaoui S. Y. Yu Z. Coulombe Y. Karam M. Busatto F. F. Masson J. Y. (2019). Arginine methylation of the DDX5 helicase RGG/RG motif by PRMT5 regulates resolution of RNA:DNA hybrids. EMBO J. 38 (15), e100986. 10.15252/embj.2018100986 31267554 PMC6669924

[B26] Nicol S. M. Bray S. E. Black H. D. Lorimore S. A. Wright E. G. Lane D. P. (2013). The RNA helicase p68 (DDX5) is selectively required for the induction of p53-dependent p21 expression and cell-cycle arrest after DNA damage. Oncogene 32 (29), 3461–3469. 10.1038/onc.2012.426 22986526 PMC3556166

[B28] Ognjanovic S. Linabery A. M. Charbonneau B. Ross J. A. (2009). Trends in childhood rhabdomyosarcoma incidence and survival in the United States, 1975-2005. Cancer 115 (18), 4218–4226. 10.1002/cncr.24465 19536876 PMC2953716

[B29] Rossi F. Centrón-Broco A. Dattilo D. Di Timoteo G. Guarnacci M. Colantoni A. (2021). CircVAMP3: a circRNA with a role in alveolar rhabdomyosarcoma cell cycle progression. Genes (Basel) 12 (7), 985. 10.3390/genes12070985 34203273 PMC8303801

[B30] Saporita A. J. Chang H. C. Winkeler C. L. Apicelli A. J. Kladney R. D. Wang J. (2011). RNA helicase DDX5 is a p53-independent target of ARF that participates in ribosome biogenesis. Cancer Res. 71 (21), 6708–6717. 10.1158/0008-5472.CAN-11-1472 21937682 PMC3206203

[B31] Sergeeva O. Zatsepin T. (2021). RNA helicases as shadow modulators of cell cycle progression. Int. J. Mol. Sci. 22 (6), 2984. 10.3390/ijms22062984 33804185 PMC8001981

[B32] Skapek S. X. Ferrari A. Gupta A. A. Lupo P. J. Butler E. Shipley J. (2019). Rhabdomyosarcoma. Nat. Rev. Dis. Prim. 5 (1), 1. 10.1038/s41572-018-0051-2 30617281 PMC7456566

[B33] Sun X. Guo W. Shen J. K. Mankin H. J. Hornicek F. J. Duan Z. (2015). Rhabdomyosarcoma: advances in molecular and cellular biology. Sarcoma 2015, 232010. 10.1155/2015/232010 26420980 PMC4569767

[B34] Tabassum S. Basu M. Ghosh M. K. (2023). The DEAD-Box RNA helicase DDX5 (p68) and β-catenin: the crucial regulators of FOXM1 gene expression in arbitrating colorectal cancer. Biochim. Biophys. Acta Gene Regul. Mech. 1866 (2), 194933. 10.1016/j.bbagrm.2023.194933 36997114

[B35] Takeda K. Ohta S. Nagao M. Kobayashi E. Tago K. Funakoshi-Tago M. (2024). FL118 is a potent therapeutic agent against chronic myeloid leukemia resistant to BCR-ABL inhibitors through targeting RNA helicase DDX5. Int. J. Mol. Sci. 25 (7), 3693. 10.3390/ijms25073693 38612503 PMC11011477

[B36] Tentler J. J. Lang J. Capasso A. Kim D. J. Benaim E. Lee Y. B. (2020). RX-5902, a novel β-catenin modulator, potentiates the efficacy of immune checkpoint inhibitors in preclinical models of triple-negative breast cancer. BMC Cancer 20 (1), 1063. 10.1186/s12885-020-07500-1 33148223 PMC7641792

[B37] Tian M. Wei J. S. Shivaprasad N. Highfill S. L. Gryder B. E. Milewski D. (2023). Preclinical development of a chimeric antigen receptor T cell therapy targeting FGFR4 in rhabdomyosarcoma. Cell Rep. Med. 4 (10), 101212. 10.1016/j.xcrm.2023.101212 37774704 PMC10591056

[B38] Wachtel M. Schäfer B. W. (2018). PAX3-FOXO1: zooming in on an “undruggable” target. Semin. Cancer Biol. 50, 115–123. 10.1016/j.semcancer.2017.11.006 29146205

[B39] Wang Z. Luo Z. Zhou L. Li X. Jiang T. Fu E. (2015). DDX5 promotes proliferation and tumorigenesis of non-small-cell lung cancer cells by activating β-catenin signaling pathway. Cancer Sci. 106 (10), 1303–1312. 10.1111/cas.12755 26212035 PMC4638002

[B40] Wang J. Han C. Wang J. Peng Q. (2023). RNA helicase DDX5-induced circPHF14 promotes gastric cancer cell progression. Aging (Albany NY) 15, 2525–2540. 10.18632/aging.204623 36996491 PMC10120908

[B41] Williamson D. Missiaglia E. de Reyniès A. Pierron G. Thuille B. Palenzuela G. (2010). Fusion gene-negative alveolar rhabdomyosarcoma is clinically and molecularly indistinguishable from embryonal rhabdomyosarcoma. J. Clin. Oncol. 28 (13), 2151–2158. 10.1200/JCO.2009.26.3814 20351326

[B42] Wu G. Xing Z. Tran E. J. Yang D. (2019). DDX5 helicase resolves G-quadruplex and is involved in *MYC* gene transcriptional activation. Proc. Natl. Acad. Sci. U. S. A. 116 (41), 20453–20461. 10.1073/pnas.1909047116 31548374 PMC6789965

[B43] Wu J. You Y. Q. Ma Y. X. Kang Y. H. Wu T. Wu X. J. (2020). DDX5-targeting fully human monoclonal autoantibody inhibits proliferation and promotes differentiation of acute promyelocytic leukemia cells by increasing ROS production. Cell Death Dis. 11 (7), 552. 10.1038/s41419-020-02759-5 32690860 PMC7371707

[B44] Xu K. Sun S. Yan M. Cui J. Yang Y. Li W. (2022). DDX5 and DDX17-multifaceted proteins in the regulation of tumorigenesis and tumor progression. Front. Oncol. 12, 943032. 10.3389/fonc.2022.943032 35992805 PMC9382309

[B46] Yang L. Lin C. Zhao S. Wang H. Liu Z. R. (2006). P68 RNA helicase mediates PDGF-Induced epithelial-mesenchymal transition by displacing axin from β-catenin. Cell 127 (1), 139–155. 10.1016/j.cell.2006.08.036 17018282

[B47] Yang Y. Fan X. Mao M. Song X. Wu P. Zhang Y. (2017). Extensive translation of circular RNAs driven by N6-methyladenosine. Cell Res. 27 (5), 626–641. 10.1038/cr.2017.31 28281539 PMC5520850

[B49] Yu Z. Mersaoui S. Y. Guitton-Sert L. Coulombe Y. Song J. Masson J. Y. (2020). DDX5 resolves R-loops at DNA double-strand breaks to promote DNA repair and avoid chromosomal deletions. Nar. Cancer 2 (3), zcaa028. 10.1093/narcan/zcaa028 33015627 PMC7520851

[B50] Yuan J. Song Y. Pan W. Li Y. Xu Y. Xie M. (2020). LncRNA SLC26A4-AS1 suppresses the MRN complex-mediated DNA repair signaling and thyroid cancer metastasis by destabilizing DDX5. Oncogene 39 (43), 6664–6676. 10.1038/s41388-020-01460-3 32939012

[B53] Zhang T. Yang X. Xu W. Wang J. Wu D. Hong Z. (2021). Heat shock protein 90 promotes RNA helicase DDX5 accumulation and exacerbates hepatocellular carcinoma by inhibiting autophagy. Cancer Biol. Med. 18 (3), 693–704. 10.20892/j.issn.2095-3941.2020.0262 33764710 PMC8330532

[B54] Zhao W. Wang Z. Sun Z. He Y. Jian D. Hu X. (2018). RNA helicase DDX5 participates in oxLDL-induced macrophage scavenger receptor 1 expression by suppressing mRNA degradation. Exp. Cell Res. 366 (2), 114–120. 10.1016/j.yexcr.2018.03.003 29522752

